# Recent Progress in the Fabrication, Properties, and Devices of Heterostructures Based on 2D Materials

**DOI:** 10.1007/s40820-019-0245-5

**Published:** 2019-02-18

**Authors:** Yanping Liu, Siyu Zhang, Jun He, Zhiming M. Wang, Zongwen Liu

**Affiliations:** 1 School of Physics and Electronics, Hunan Key Laboratory for Super-Microstructure and Ultrafast Process, Central South University, 932 South Lushan Road, Changsha, 410083 Hunan People’s Republic of China; 2 State Key Laboratory of Electronic Thin Films and Integrated Devices, University of Electronic Science and Technology of China, Chengdu, 610054 People’s Republic of China; 3 School of Chemical and Biomolecular Engineering, The University of Sydney, Sydney, NSW 2006 Australia

**Keywords:** Two-dimensional (2D) materials, 2D heterostructures, Charge and magnetotransport, Electronic and optoelectronic devices

## Abstract

With a large number of researches being conducted on two-dimensional (2D) materials, their unique properties in optics, electrics, mechanics, and magnetics have attracted increasing attention. Accordingly, the idea of combining distinct functional 2D materials into heterostructures naturally emerged that provides unprecedented platforms for exploring new physics that are not accessible in a single 2D material or 3D heterostructures. Along with the rapid development of controllable, scalable, and programmed synthesis techniques of high-quality 2D heterostructures, various heterostructure devices with extraordinary performance have been designed and fabricated, including tunneling transistors, photodetectors, and spintronic devices. In this review, we present a summary of the latest progresses in fabrications, properties, and applications of different types of 2D heterostructures, followed by the discussions on present challenges and perspectives of further investigations.

## Highlights

- The controllable fabrication methods, the unique properties, and relative applications of 2D heterostructures were summarized.
- The generation and detection of interlayer excitons in 2D heterostructures with type II band alignment indicate a longer lifetime and larger binding energy than intralayer excitons.
- The advances in magnetic tunneling junctions based on 2D heterostructures can be applied in spintronic devices to realize spin filtering.

## Graphical abstract

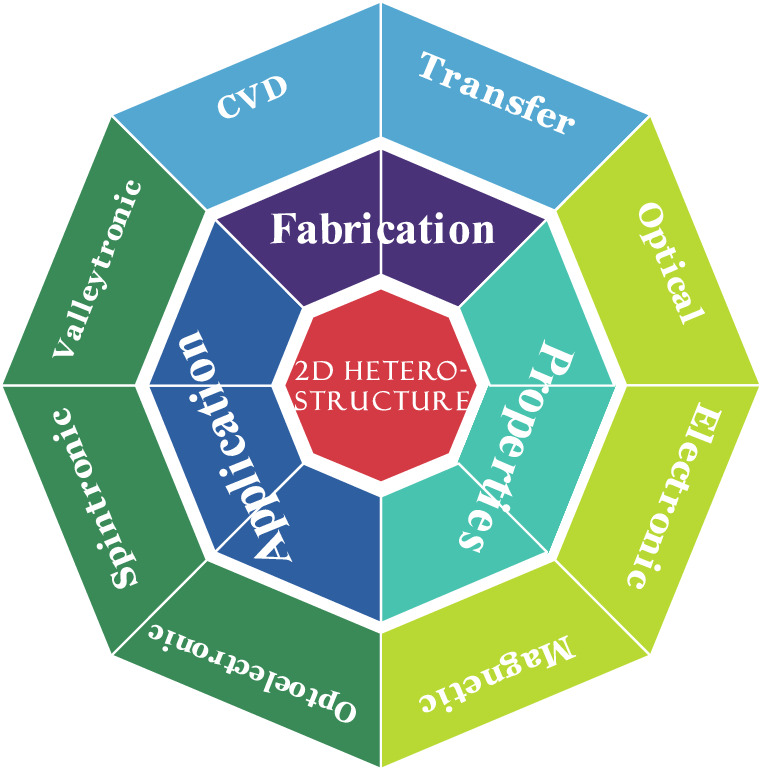

## Introduction

Since the successful preparation of graphene in 2004 [1], the properties and applications of two-dimensional (2D) materials have attracted much attention. 2D materials can be divided into single-element 2D materials (such as graphene, black phosphorus (BP), silylene, germanene, etc.) and compound 2D materials (TMDs, hBN, TMCs, III–V group elements, compound semiconductor, etc.) [2]. Compared to bulk materials, layered 2D materials possess many peculiar properties that are strongly related to their number of layers. For example, the band structure at the Dirac point of a monolayer of graphene exhibits a linear dispersion relation, which is quite different from the parabolic band structure of double-layered grapheme [3]. And for black phosphorus (BP), the bandgap displays an evident redshift as the number of layers increases [4]. Besides, the direct-to-indirect band gap transition has been demonstrated in TMD semiconductors when it changes from single layer to multilayer [5]. 2D thin-layered materials have been considered as promising building blocks for the next generation of electronic and optoelectronics devices due to their extraordinary properties. In particular, 2D atomically thin structures are immune from the short channel effect and their mechanical strength allows for integration into flexible and wearable circuits [6, 7].

However, a number of issues limit the application of single 2D materials. Specifically, the direct deposition of metal electrodes on 2D semiconductors during device fabrication results in a high contact resistance due to the Schottky barrier [8–10]. Also, the intralayer excitons generated in single 2D semiconductor materials are hard to manipulate due to their short lifetime [11], which restricts their applications in exciton devices. Furthermore, hBN and most insulated 2D materials are not suitable to be applied in devices alone, and BP is easily oxidized when exposed to air.

Therefore, the idea of that 2D materials can be assembling 2D materials into heterostructures was put forward [12], and many novel properties of these heterostructures have been discovered [13–19]. Heterostructured 2D materials can be divided into two categories: the vertically stacked heterostructures and the epitaxial grown planar heterostructures. In this paper, we focus on the fabrication methods, the properties, and the applications of 2D heterostructures in these two types.

## Fabrications of 2D Heterostructures

### Deterministic Transfer Method

**Fig. 1:**
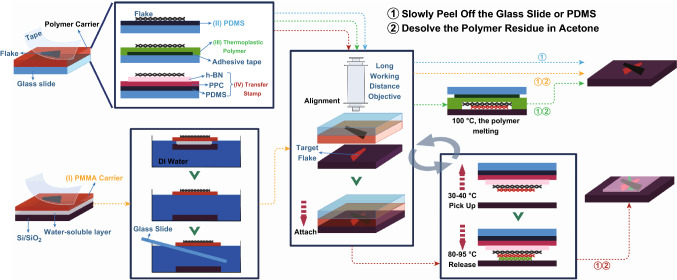
The deterministic transfer method. The yellow, blue, green, and red dashed lines correspond to the process of transfer using polymer carriers of types I to IV, respectively, and in the end a bare heterostructure or a multilayer heterostructure covered by hBN will be fabricated. (Color figure online)

The layered 2D materials prepared through mechanical exfoliation and chemical vapor deposition can be transferred onto different substrates at a desired location. This usually requires a long working distance optical inspection system in combination with an XYZθ direction micro-manipulator for accurate placement and certain polymer layers as a transfer medium. There are four types of polymer carriers: (I) PMMA/sacrifice layer [12, 20], (II) PDMS [21, 22], (III) thermoplastic polymer [23], and (IV) hybrid stamp composed of PDMS/PPC (or PC, PMMA)/hBN [24]. The transfer processes are slightly different from each other as schematically illustrated in Fig. 1.

Step 1: The polymer carriers are spin-coated layer by layer onto a Si/SiO_2_ substrate (mainly for type I transfer media) or a glass slide and then mechanically exfoliate thin flakes of 2D nanostructures onto the carriers; Step 2: mount the glass slide on the micro-manipulator directly or after the wet transfer process (mainly for type I carriers, the PMMA with flakes is attached to a glass slide when released from the Si/SiO_2_ substrate through desolating the sacrificial layer) and then align the flakes on the polymer carriers with the target material prepared on existing substrate into the desired location and orientation under microscope with micro-manipulate arms, and then the glass slide was lowered down until the two flakes make contact to form the van der Waals heterostructure; Step 3: directly pick up the target flakes and combine with another layered material to form multi-heterostructure (for type IV carriers) or release the heterostructure by slowly lifting the glass slide and remove the residual polymer in acetone solution. (For type ΙΙΙ and type IV transfer media, heating substrates are needed to release the stacking.)

The comparison between the different deterministic transfer methods is presented in Table 1 [25]. The cleanness is mainly determined by whether the interfaces in the heterostructure are exposed to the polymer. And the easiness and speed are dependent on the number of steps and specific procedures such as spin coating, heating, and wet transferring, which will increase the complexity and slow down the speed to some extent. The PDMS dry transfer is considered the easiest and quickest method, as the progress does not involve spin coating, wet transfer, or heating process. PDMS films have become commercialized commodities, and their viscosity is strongly related to the speed when being peeled off [26]. Meanwhile, the pickup method remains the best way to assemble multi-heterostructure with no residual polymer at interfaces. In conclusion, the deterministic transfer method has high flexibility in fabricating various heterostructures, but its drawbacks are obvious. It is inevitable to cause polymer residue at the interface or on the surface, and the 2D material samples may be destroyed or got wrinkled during the transfer process. Another challenge of the deterministic transfer method is the control of the stack orientation.

**Table 1:** Comparison between the different deterministic placement methods^1^. Reprinted with permission from Ref. [25]

Carrier type	Cleanness	Easiness	Speed
PMMA/sacrifice layer	▲▲▲	▲▲▲	▲▲▲
PDMS	▲▲▲	▲▲▲▲▲	▲▲▲▲▲
Thermoplastic polymer	▲	▲▲	▲▲▲
PDMS/PPC (or PC, PMMA)/hBN	▲▲▲▲▲	▲	▲▲

^1^The numbers of symbol ▲ indicate the degrees in cleanness, easiness and speed

### Chemical Vapor Deposit (CVD) Growth

CVD synthesis is a bottom-up strategy for the preparation of vertical 2D heterostructures and is able to realize the growth of planar multi-junction heterostructures. In order to obtain an atomically sharp heterojunction and a clean interface in vertically stacked heterostructures, as well as achieve scalable and controllable fabrication of both vertical and planar heterostructures, various approaches have been explored to realize CVD growth of heterostructures.

#### The One-Step CVD Method

**Fig. 2:**
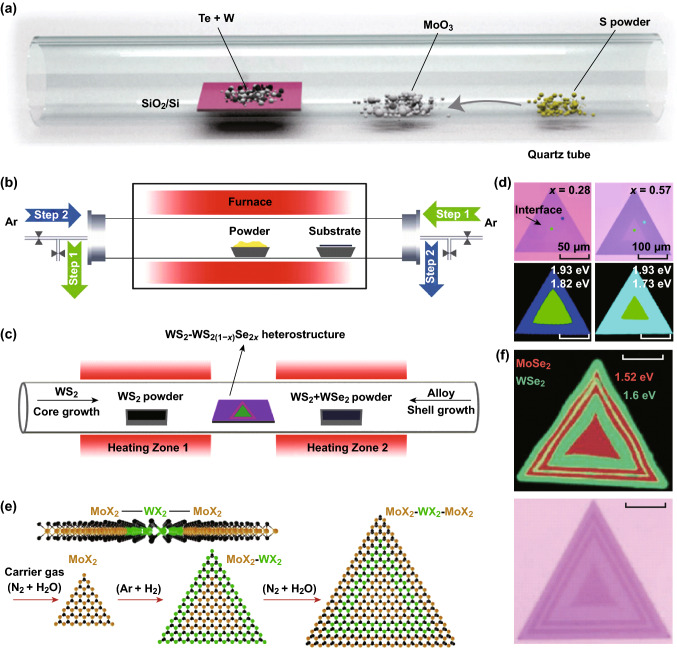
Chemical vapor deposit (CVD) growth. **a** The diagram of the synthesis of MoS_2_/WS_2_ heterostructures through one-step CVD method. S powder is placed at the upstream, and a wafer with mixed powder of W and Te is put downstream. Reprinted with permission from Ref. [27]. **b** Multi-step CVD growth of realized through direction-switchable carrier flow and cooling process. Reprinted with permission from Ref. [32]. **c** the modulable growth of WS_2_ − WS_2(1−*x*)_Se_2*x*_ (0 < *x *≤ 1) monolayer lateral heterostructures using dual heating quartz tube and **d** the optical picture and PL intensity mappings of heterostructures with different *x*. Reprinted with permission from Ref. [30]. **e** The principle of one-pot synthesis strategy and **f** the optical picture and PL intensity mappings of the hetero-superlattice indicating the sharp interlines. Reprinted with permission from Ref. [31]

The schematic diagram of Fig. 2a shows the processes of this method, which is suitable for preparing heterostructures with the component materials containing the same elements. Gong et al. [27] successfully fabricated both vertical and planar MoS_2_/WS_2_ heterostructures by this one-step CVD method that demonstrated the in-plane epitaxial growth at a lower temperature of 650 °C, as the provided energy was not enough for the nucleation of WS_2_ on the surface of MoS_2_, but at a higher temperature of 850 °C vertically stacked structure could be formed as the van der Waals heterostructure was more thermodynamically stable.

#### The Two-Step CVD Method

This is a more commonly adopted approach using the as-grown layered crystals as a substrate for the second layer. Such procedures were also adapted for both the vertically stacked and lateral heterostructures that are mainly affected by the rate of gas flow and the synthesis time. Li et al. [28] reported the growth of 2D GaSe/MoSe_2_ heterostructure by this two-step CVD method. Such MX/MX_2_ vertical heterostructures exhibit incommensurate superstructures because of the large lattice misfit between the two layers. In addition, the synthesis of a stacked TMD/hBN heterostructure was also realized by using the Ni–Ga alloy and Mo foil as the substrate without any intermediate operations. In such a case, the Ni–Ga alloy promoted the formation of the hBN honeycomb lattice, while the Mo foil was a source of Mo [29].

#### The Multi-step CVD Method

By switching the direction and modulating the components of gas flow [30–32], the multi-step CVD method was developed (Fig. 2b). This approach makes the boundary of heterojunctions sharper and enables the sequential growth of the multi-junction heterostructure. Biyuan Zheng et al. [30] reported an efficient method to grow modulable WS_2_ − WS_2(1−*x*)_Se_2*x*_ (0 < *x *≤ 1) monolayer planar heterostructure (Fig. 2c) with tunable band alignment (Fig. 2d). Using the dual heating furnace, the switching of the synthesis from a pure WS_2_ growth to a WS_2(1−*x*)_Se_2*x*_ (0 < *x* ≤ 1) alloy formation can be controlled by changing the direction and the temperature of the Ar gas flow. The parameter *x* is modulated by the ratio of the mixed WS_2_/WSe_2_ powders.

Moreover, the control of growth can also be realized by changing the components of the gas flow. Sahoo et al. [31] reported the one-pot synthesis strategy by placing two precursors (MoX_2_ and WX_2_ mixed powders) in the same boat at the heating zone, while the substrate was held at a lower temperature. The mechanism is vividly illustrated in Fig. 2e: under the N_2_ + H_2_O (g) gas flow, only the growth of MoX_2_ was promoted. When the gas flow was switched to the Ar + H_2_(5%) gas flow, only the growth of WX_2_ was allowed. Reversing the gas flow would terminate the current reaction. Figure 2f shows the optical images and the Raman intensity maps at 240 and 250 cm^−1^ revealing the well-defined spatial distribution and sharp interface of the MoSe_2_ and WSe_2_ domains in the multi-junction heterostructure. Compared with the one-step and the two-step CVD methods, the multi-step CVD method is more flexible and controllable that enables the possibility of creating spatially selected optoelectronic devices due to the separation of electrons and holes into different materials.

## Properties

### Band Alignment

**Fig. 3:**
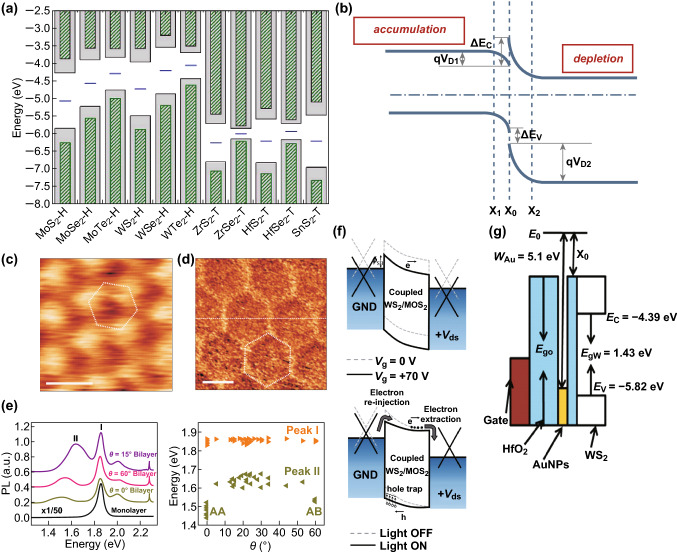
Band alignment and band engineering methods. **a** Theoretical positions of VBM and CBM of VIB- and IVB-TMDs calculated by PBE-SOC. Reprinted with permission from Ref. [33]. **b** Schematic diagram of band bending at the interface, forming a notch on the accumulation side and a peak on the depletion side. **c**, **d** STM measurements of hBN/graphene heterostructure stacked with the different rotation angle of 5° (**c**) and 0° (**d**). Reprinted with permission from Ref. [41]. **e** PL spectra of MoS_2_ monolayer and bilayers with different twist angles, where peak I is related to the direct transition at *K*_−_ valley and peak II may be associated with the indirect band gap. Reprinted with permission from Ref. [41]. **f** The principle of photogating band alignment engineering. Reprinted with permission from Ref. [42]. **g** The band structure of a floating-gate device based on monolayer WS_2_. Reprinted with permission from Ref. [43]

The band alignment is the fundamental property of 2D heterostructures. Most charge transport behavior and illuminance properties are originated from the band structures, especially the band alignment. The band energy can be calculated in theory based on the first principles and measured through μ-XPS. Figure 3a [33] shows the band alignments of a monolayer semiconducting TMDs and monolayer SnS_2_ calculated by Perdew–Burke–Ernzerhof (PBE) with spin–orbit coupling (SOC). The results indicate that, for the monolayer TMDs in MX_2_ form, the energy of the conduction band minimum (CBM) and the valence band maximum (VBM) increases with the atomic number of X. Thus, when two different materials with sizable band gaps get combined into a heterostructure, there will be three types of I–III band alignments without considering the band bending at the interface, which are called straddling gap, staggered gap, and broken gap, respectively. The type I band alignment contributes to the fast recombination of electrons and holes that allows them to be used in luminescent devices, such as light-emitting diodes (LEDs) [34, 35]. The type II band alignment can facilitate the effective spatial separation of electrons and holes, prolonging the lifetime of interlayer excitons and making them a good candidate for the application in electron–hole separators and related optoelectronic devices. The type III band alignment allows the band-to-band tunneling (BTBT) effect of carriers and enables the operation of the tunnel field effect transistors (TFET). Compared with the type II band alignment, the speed of the transportation of the electrons and holes in type III heterostructures is much faster that results in a large number of electrons and holes separating into different layers of the material, so the heterojunction displays semi-metallic features generating a strong built-in electric field that makes the type III heterostructures ideal for new-generation thermal photovoltaic cells.

Interestingly, when taking the band bending at the interface into consideration, the band structure of the heterostructures presents more novel properties. For example, when two semiconductors form a PN junction with type I band alignment (Fig. 3b), the VB and the CB on the different sides will bend in the opposite directions, forming a notch and a peak, respectively. There is none symmetry in Δ*E*_C_ and Δ*E*_V_ values which will cause different potential barriers for electrons and holes.

In addition, the band structure of a 2D heterostructure can be affected by many factors. Firstly, the quality of the interface is very important. For example, the interface defects and impurities can cause the defect or impurity energy level; and intrinsic metal-induced gap states (MIGS) or extrinsic disorder induced gap states (DIGS) at the interface would lead to the occurrence of Fermi-level pinning effect [36]. Bampoulis et al. [37] demonstrated that the subsurface metal-like defects in MoS_2_/metal heterostructures could hugely decrease the Schottky barrier height (SBH) that attributed to strong Fermi-level pinning at the defects. On the other hand, a weak Fermi-level pinning led to the SBH modulation by electrical gating [38], and a very weak Fermi-level pinning has been observed at the graphene/TMD interfaces. A shift of about 120 mV was observed between the spectra of scanning tunneling spectroscopy (STS) of flakes residing on single-layer graphene (SLG) and bilayer graphene (BLG) substrates, which is equal to the difference in the work function of SLG and BLG. Secondly, the lattice constants of the materials and their stake orientation are also important factors. When two 2D materials with similar lattice constants form heterostructures, such as graphene/hBN [39] and SnS_2_/MoS_2_ [40], a periodic Moiré pattern will be formed. Figure 3c, d shows the STM measurements on graphene/hBN heterostructure with a rotation smaller (Fig. 3d) and larger (Fig. 3c) than 1°. The later one displays a nearly same spatial periodic behavior exhibiting a much larger Moiré pattern due to the high Young modulus of graphene. Such deformation of lattice structure results in a much stronger van der Waals interaction and causes extra periodic potential in graphene that adjusts its band structure. Moreover, the indirect band gap varies appreciably with the stacking orientation [41]: the largest redshift for AA-(twist angle of 0°) and AB-(twist angle of 60°) stacked bilayers and a significantly smaller but constant redshift for all other twist angles (Fig. 3e).

However, adjusting the band alignment through the above structural factors is complex and inflexible. The band structure of heterojunction can also be externally controlled by an electric field, magnetic field and light field [44]. The band engineering regulated by the gate voltage leads to the transitions in 2D heterostructures between band alignments of type I–III. Particularly, graphene has a unique linear dispersion relationship with a finite DOS; thus, the Fermi level and work function of graphene are gate modulable, making graphene widely used as the contact between the electrodes and the channel of the 2D material to reduce the contact resistance (*R*_c_). What’s more, band structure can also be optically modulated to behave like a photogate, a phenomenon looking like the addition of a local gate (Δ*V*_g_) to the device when under illumination [45]. The band diagram of a photodetector with the WS_2_/MoS_2_ hetero-bilayer as a light absorption layer and the graphene as the electrode contact is shown in Fig. 3f [42]. Under illumination, the accumulation of holes at the region acts as a positive gate on the source graphene electrode, effectively raising the Fermi level of the graphene electrode and lowering the SBH. Figure 3g [43] shows a typical band structure of floating-gate devices with an Au floating-gate layer to form energy well to trap the charges.

### Charge Transport Properties in 2D Heterostructures

Different band alignments result in a variety of charge transport characteristics accompanied by the process of energy transfer. On the one hand, the single-particle transportation, including interlayer tunneling effect and charge trapping phenomena, has been widely reported and novel devices based on those transportation mechanisms have been fabricated that exhibit low energy-assumption with high performance; on the other hand, the many-body transport and the separation of electron–hole pairs in 2D heterostructures have also been theoretically predicted and practically demonstrated [11], which enable the prospect of broad applications in optoelectronic field [46–50].

#### Single-Particle Transports

**Fig. 4:**
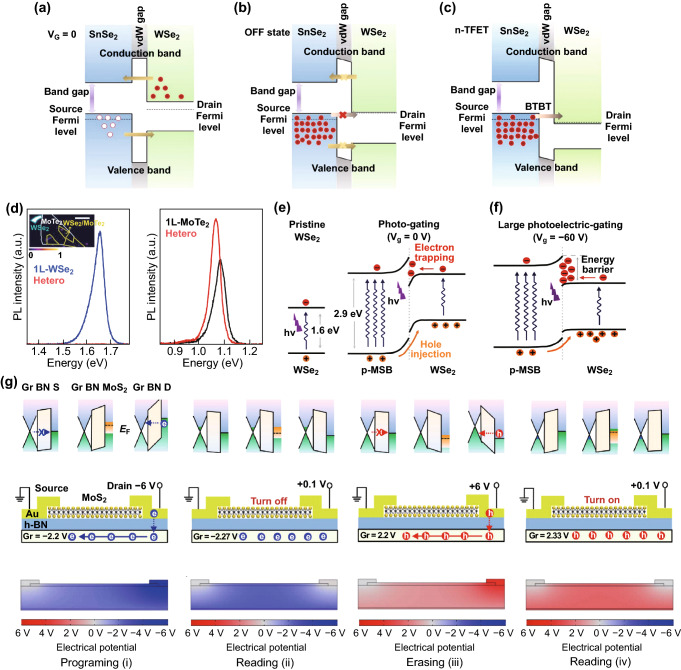
The single-particle transport caused by different band structures. **a**–**c** The electronic modulation of BTBT in a TFET based on 2D SnSe_2_/WSe_2_ heterostructure. Reprinted with permission from Ref. [52]. **d** PL measurement of a stacked WSe_2_/MoTe_2_ heterostructure with proper annealing, where the blue, black, and red lines correspond to the PL intensity of specific energy (eV) from the isolated WSe_2_ region, isolated MoTe_2_ region, and hetero-stacked region, respectively. Reprinted with permission from Ref. [53]. **e**, **f** The diagram of the charge transferring in p-MSB/WSe_2_ heterostructure under illumination. Reprinted with permission from Ref. [54]. **g** The band alignment, charge transportation, and the distribution of the potential in the device. Reprinted with permission from Ref. [39]. (Color figure online)

The ultra-narrow channel length and atomically sharp interfaces can realize band-to-band tunneling (BTBT) effect by electrostatic gating to avoid deprivation of band-edge sharpness resulted from chemical doping [51]. As shown in Fig. 4a–c [52], when applying a small *V*_g_ (Fig. 4b), the CBM of WSe_2_ is higher than the VBM of SnSe_2_. The electrons in SnSe_2_ cannot tunnel into WSe_2_, but diffusion of charges occurs at the interface, corresponding to channel current and high resistance state, respectively, when applied with a positive and negative bias voltage. This corresponds to the off state of the device. When keeping on increasing the *V*_g_ while the CBM of WSe_2_ is set below the VBM of SnSe_2_ (Fig. 4c), a tunneling window is opened, such that an interlayer tunneling can flow from SnSe_2_ to WSe_2_. In type I band alignment, the layer with a wide bandgap will cause spatial confinement of electrons and holes in the “well” layer with a narrow bandgap [53] that causes a strong PL in a trapping layer and a quenched PL in the other (Fig. 4d).

When taking the band bending into consideration, the interfacial charge trapping can be seen in the PN junction of type II band alignment. Cai et al. [54] developed an electric-gating switchable photodetector based on p-MSB/WSe_2_ by epitaxial growth of 2D van der Waals, in which the p-MSB serves as a light absorber, while in the p-MSB/WSe_2_ heterostructure an interfacial energy barrier and a band bending between the lowest unoccupied molecular orbital of p-MSB and the CBM of WSe_2_ (Fig. 4e) enable unidirectional carrier injection. When a negative *V*_g_ is applied (Fig. 4f), the Fermi level of WSe_2_ shifts downward, leading to an increase of the interfacial energy barrier that enhances the interfacial charge trapping process. It is believed the interface quality is the key to avoid the on-state current decreasing under such plasma treatment.

Another way to obtain charge trapping is to place a floating-gate layer, which is usually sandwiched with the tunneling and blocking dielectric layers, between the channel and control gate as a charge trapping layer [43, 55–60]. Figure 4g [61] shows the charge transport in the floating-gate structure with graphene as the trapping layer and monolayer MoS_2_ as the channel. When a − 6 V (+ 6 V) drain bias is applied, a large potential difference between the drain and graphene, and a nearly equal potential of the source electrode and graphene are generated. As a result, the electrons (and holes) are able to tunnel through the hBN layer at the drain electrode side and are trapped in the floating-gate layer, so are unable to tunnel back to the source electrode or the MoS_2_ channel.

#### The Generation of Interlayer Excitons

**Fig. 5:**
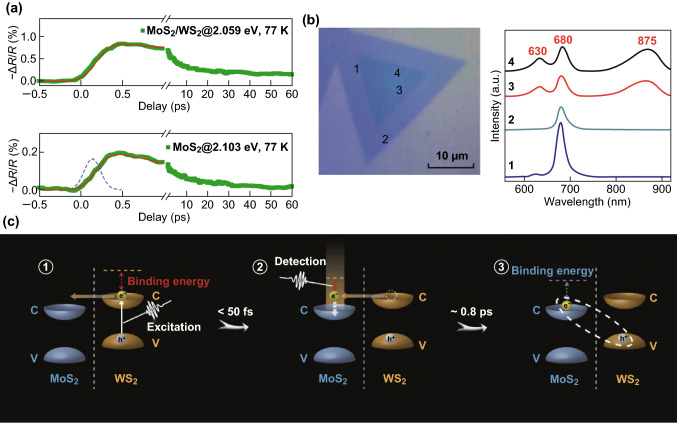
The generation of interlayer excitons. **a** The transient absorption measurements of WS_2_ A-exciton resonance in the MoS_2_/WS_2_ heterostructure and B-exciton resonance in an isolated MoS_2_ monolayer. The dynamic evolution signal is obtained by convoluting the instrument response function (blue dashed line). **b** PL spectra detected from the 1–4 region of a vertically stacked WS_2_/MoS_2_ heterostructure, and numbers 1 and 2 correspond to the MoS_2_ monolayer and number 3 and 4 regions are hetero-stacked bilayer. Two additional peaks at 630 and 875 nm indicate the generation of interlayer excitons. Reprinted with permission from Ref. [27]. **c** The two processes of the interlayer excitons generation. Reprinted with permission from Ref. [62]. (Color figure online)

For the type II band alignment, the CBM and VBM are in different layers that lead to the spatial separation of electrons and holes. The ultrafast separation of electron–hole pairs could be detected by the pump–probe technique [48, 62, 63]. In the WS_2_/MoS_2_ heterostructure, after the MoS_2_ monolayer is directly pumped the photo-induced signal will appear instantaneously. The experimentally observed signal in the heterostructure shows a rising time that is shorter than 50 fs (Fig. 5a).

The detailed charge separation processes in the heterostructure are presented in Fig. 5c [62]. After the excitation of the WS_2_(MoS_2_) monolayer, the electrons (holes) transferred from the CBM (VBM) of WS_2_ (MoS_2_) monolayer to the conduction (valence) bands just above (below) CBM (VBM) of the MoS_2_(WS_2_) monolayer within 50 fs, generating a hot interlayer exciton with a relatively long distance. The hot excitons then reorganized and dissipated the excess energy to form the tightly bound interlayer excitons within 800 fs. The existence of the final tightly bound interlayer excitons could be distinctly demonstrated in photoluminescence spectra [62, 64] as shown in Fig. 5b [27]. The photoluminescence signal was taken from number 1 and 2 regions corresponding to the A-exciton resonances in the monolayer MoS_2_. But the signals from the heterostructure regions displayed exciton resonances in MoS_2_ and WSe_2_, leading to an additional interlayer exciton peak. The exciton photoluminescence signals of MoS_2_ and WSe_2_ were quenched in the heterostructure due to the charge transfer process.

### The Properties of Excitons in 2D Heterostructure

**Fig. 6:**
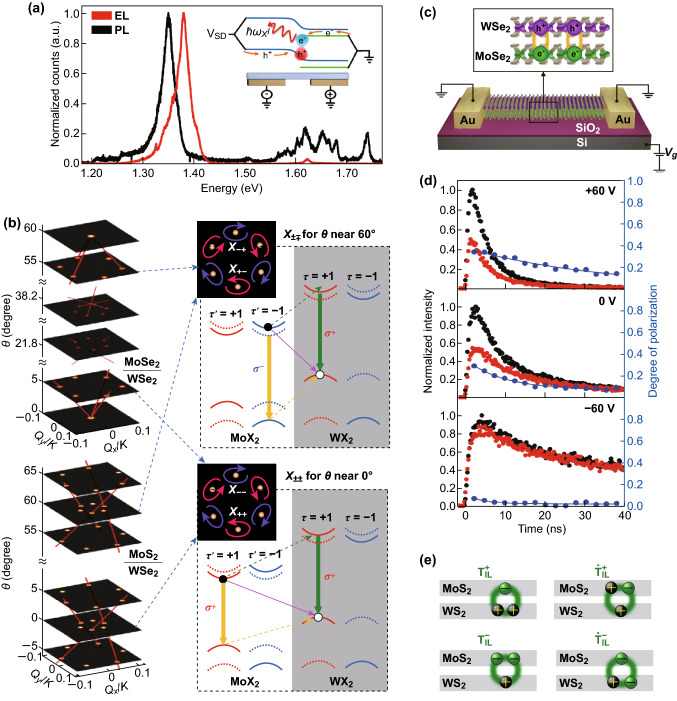
The properties of excitons in 2D heterostructures. **a** EL spectra of the MoSe_2_/WSe_2_ vertical heterostructure, a small intralayer signal at 1.62 eV was detected, and the inset illustrates the device structure. (Blue and green lines represent the WSe_2_ and MoSe_2_, respectively.) Reprinted with permission from Ref. [66]. **b** The optical selection rules in a MoX_2_/WX_2_ heterostructure with twist angle *θ* of 0° and 60°, respectively. Reprinted with permission from Ref. [17]. **c**, **d** Time-resolved PL (**d**) of WSe_2_/MoSe_2_ heterostructure (**c**) after *σ*+ pulsed laser excitation, black dots and red dots corresponding to the co-polarized emission (*σ*+) and cross-polarized emission (*σ*−), and the blue line shows the degree of polarization, which is tunable by the gate voltage. Reprinted with permission from Ref. [67]. **e** The trions composed in different ways existing in the MoS_2_/WS_2_ bilayer. Reprinted with permission from Ref. [68]. (Color figure online)

The interlayer excitons hold the prolonged lifetime [50] and large binding energy which is strongly related to the distance between the two layers in vertical heterostructures. Simone Latini et al. [65] developed a QEH (quantum electrostatic heterostructure) model based on the MoS_2_/*x*-hBN/WSe_2_ vertical heterostructures. (The parameter *x* is the number of hBN layers.) The binding energy of the interlayer excitons is calculated up to 0.3 eV and decreases with the increasing parameter *x*, indicating a stable existence of the interlayer excitons at room temperature. But the weak oscillator strength and momentum-indirect nature make it challenging to directly observe the interlayer excitons by resonant optical excitation [66]. Figure 6a shows the spectra of electroluminescence (red line) and photoluminescence (black line) for lateral p–n junctions based on a vertical MoSe_2_–WSe_2_ hetero-bilayer. The inset picture illustrates the electrode contacts of each layer. When applying a forward *V*_sd_, the carriers would be injected in and recombine at the edge of the hetero-bilayer (orange arrows). It turned out that the photocurrent amplitude from the interlayer exciton was about 200 times smaller than that of the resonant excitation of the intralayer exciton.

Interlayer excitons also have ideal valley-contrast physics. Figure 6b [17] shows the schematic of the interlayer exciton recombination in the *K*_±_ valley in a MoS_2_/WSe_2_ heterostructure with the twist angle of 0° and 60°. After circularly polarized light excites intralayer excitons in the *K*_+_ valleys of MoS_2_ and *K*_+_ valleys of WSe_2_, a fast interlayer charge hopping forms the interlayer exciton in the *K*_+_ valley. The solid (dashed) arrows denote the dipole transition (interlayer hopping). Rivera et al. [67] observed long valley lifetime and valley drift–diffusion of host interlayer excitons in MoSe_2_–WSe_2_ heterostructures with small twist angles (Fig. 6d). The valley polarization is greatest at + 60 V and highly suppressed at − 60 V. The valley polarization lifetime increases with *V*_g_, reaching 39 ± 2 ns at + 60 V, as determined by fitting a single exponential decay. The extraordinary valley-contract properties allow the possibility of the excitonic optoelectronic circuit to switch the valley functionalities and provide a platform for investigating excitonic superfluidity and condensation.

In addition to intralayer and interlayer excitons, there is the theoretical existence of trions in 2D heterostructures. Figure 6e shows the different kinds of combinations of trions in a MoS_2_/WS_2_ heterostructure. Thorsten Deilmann et al. [68] calculated the interlayer excitation in a MoS_2_/WS_2_ heterostructure and predicted the existence of bound interlayer trions below the neutral interlayer. The binding energies are 18 and 28 meV for the positive and negative interlayer trions with both electrons/holes located on the same layer.

### Magnetic Properties in 2D Heterostructures

**Fig. 7:**
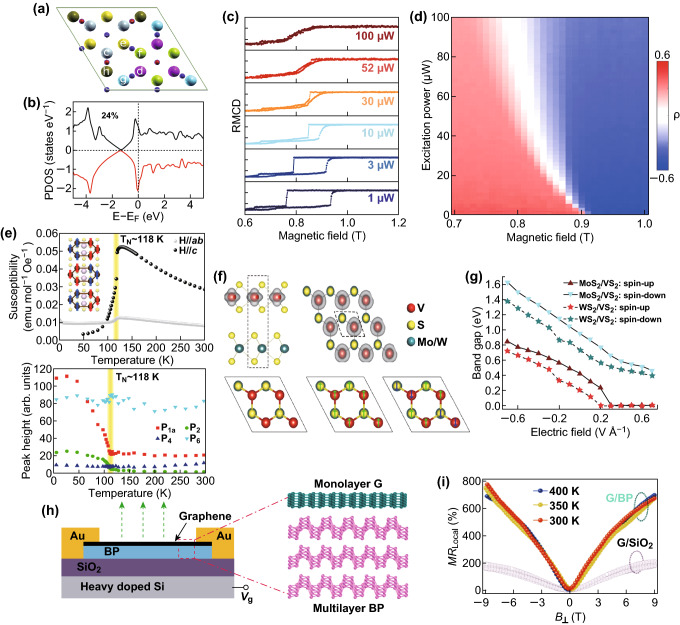
Magnetic properties of 2D heterostructures. The broken lattice with six sublattices of graphene on the EuO **(a)** and the calculated spin polarization in graphene layer (**b**). Reprinted with permission from Ref. [74]. The RMCD (**c**) and polarization ρ (**d**) of WSe_2_/CrI_3_ heterostructure under sweeping external magnetic field and different excitation power. Reprinted with permission from Ref. [75]. **e** The transition of several PL peaks and the magnetic susceptibility along the out-of-plane direction (black) in FePS_3_ as the temperature increases. Reprinted with permission from Ref. [77]. The spin density and possible magnetic ground state (NM, FM, AFM) of XS_2_/VS_2_ heterostructures (**f**) and the dependence of band gap (**g**) and electrical field, and the calculated magnetic moment and relative energy is listed in the picture. Reprinted with permission from Ref. [78]. Schematic diagram of the device based on graphene/multilayer BP heterostructure (**h**) and the huge MR (**i**) compared with graphene on SiO_2_ substrate. Reprinted with permission from Ref. [79]

For the low-dimensional magnetic materials, the coercivity, saturation magnetization, Curie temperature (*T*_C_), and other magnetic parameters of the materials are related to the number of layers and grain size. Therefore, 2D materials usually present magnetic properties that differ from its bulk form. The application of 2D magnetic materials in heterostructures is of great significance for the study of spintronics, valleytronics, and electromagnetics [69]. The magnetic properties of 2D heterostructures are significant for the external ways to control (gate) the propagation of spin and valley (polarized) currents at room temperature. The massive theoretical calculations and experimental results indicate that by assembling 2D materials (graphene or TMDs) with ferromagnetic materials into a van der Waals heterostructure [70], a large magnetic exchange field can be generated at the interface and thus the regulation of the spin and valley pseudospin in 2D materials can be realized [71–73]. The existence of the magnetic exchange field can amplify the effect of the external magnetic field, which originates from the proximity effects of the heterojunction [74]. Figure 7a shows the sublattices of graphene on EuO represented with different colors and letters. Due to the existence of the EuO substrate, the two sublattices of isolated graphene break into six folders as shown in Fig. 7b. Such structural change resulting from the proximity effect will enhance the magnetic moment of surface Eu atoms, causing variable spin polarizations on the graphene sublattices with a calculated spin polarization of about 24% in average, and change the band structure of graphene. The recent research suggests that such magnetic exchange field (MEF) can be tuned over a range of 20T by small changes in the laser excitation power [75]. Figure 7c shows the magnetization of CrI_3_ in a monolayer WSe_2_/multilayer CrI_3_ heterostructure probed via reflection magnetic circular dichroism (RMCD) as a function of the external magnetic field at different excitation light power. The RMCD exhibits a very similar power-dependent hysteresis loop behavior. Such opto-magnetic effect enables the power-switchable valley properties as illustrated in the PL spectra in Fig. 7d, where the polarization ρ (defined as (*I*_+_ − *I*_−_)/(*I*_+_ − *I*_−_), with *I*_±_ being the PL peak intensity excited by *σ*_±_ polarized laser) flips in sign at certain external magnetic field with the increasing of the excitation power.

The search of suitable 2D magnetic materials remains a challenge at the moment. The CrI_3_ mentioned above is layered magnetic material whose magnetic properties are strongly related to the number of layers [76]. Besides, a new class of magnetic heterostructure materials, called transition metal phosphorus trichalcogenides (TMPS, TM = V, Mn, Fe, Co, Ni, or Zn), could also be easily exfoliated and their magnetic ground state is strongly depended on the TM element. An Ising-type antiferromagnetic ordering from bulk to the monolayer has been reported for FePS_3_, a TMPS material. The Raman peaks (Fig. 7e) show a transition at the Neel temperature (*T*_N_) of 118 K [77].

The *T*_C_ of ferromagnetic materials in the above heterostructures is too low for them to be applied at room temperature. Current theoretical calculations have shown that XS_2_/VS_2_ heterostructures are ferromagnetic and are expected to have ultrahigh *T*_C_ [78]. Figure 7f shows the optimized configurations, local magnetic arrangements, magnetic moments and energy relative to that of the ferromagnetic configuration for non-magnetic (NM), ferromagnetic (FM), and antiferromagnetic (AFM) states, respectively. Clearly, the FM state is the most stable one for the two heterostructures. The XS_2_/VS_2_ heterostructures have the FM ground states in theory, and their *T*_C_ can be calculated by the following equation:

$$\gamma k_{\text{B}} T_{\text{C}} {\ominus }/2 = E_{\text{AFM}} - E_{\text{FM}}$$

where γ is the dimension of the XS_2_/VS_2_ system, *k*_B_ is the Boltzmann constant, and *E*_AFM_ and *E*_FM_ are the energies of the unit-cell system with AFM and FM coupling, respectively. The obtained very high *T*_c_ values are 485 K for the MoS_2_/VS_2_ heterostructure and 487 K for the WS_2_/VS_2_ heterostructure. Interestingly, a semiconductor–metal transition occurred under the modulation of the external electric field (Fig. 7g), indicating a potential for generating pure spin-polarized currents. Moreover, many other 2D magnetic materials have been studied experimentally or theoretically as shown in Table 2. Most of the 2D magnetic materials are in the form of MX (M = Cr, Co, V, Mo, Mn, etc., and X = C, O, S, Se or N) or MAX (M = Cr or Fe; A = Ge, Si, Al, Sn, etc., and X = Te), and most of them are semiconductors.

**Table 2:** The calculated or experimental properties of different 2D magnetic materials

Material	*T*_c_ (K)	Saturation magnetizations (μ_B_/unit)	Band gap (eV)	Exchange parameters	Kerr rotation angle	References
VSe_2_ ML	> 330 K	15	55 m (15 K)	–	–	[80]
Fe_3_GeTe_2_	207 K (bulk) 130 K (ML)	–	–	–	–	[81]
TL Fe_3_GeTe_2_]	~ 300 K (*V*_g_ = 1.75 V)	1.8	–	*J*_*ij*_ = 10 mV (Heisenberg model)	–	[82]
*CrC ML	> 330 K	8	2.85	*J*_1_ = 7.4 meV *J*_2_ = 14.7 meV (Heisenberg model)	–	[83]
*MnO_2_ ML	140 K 210 K (strained)	3 (< 75 K)	3.41	*J *= 1.72 meV (Ising model)	–	[84]
*Co_2_S_2_ ML	> 404 K		–	*J*_1_ = 58.7 meV *J*_2_ = 15.8 meV (Ising model)	–	[85]
GdAg_2_	85 K	5 (5 K)	–	–	–	[86]
*MnS_2_ ML	225 K, 330 K (strained)	3	0.69	–	–	[87]
*MnSe_2_ ML	250 K, 375 K (strained)	3	0.01	–	–	[87]
CrI_3_	61 K (bulk) 45 K (ML) AFM (BL)	3	–	–	5 ± 2 mrad at *μ*_oH_ = 0 T	[76]
*MoS_2_/VS_2_ HS	485 K	14.6	Tunable	–	–	[51]
*WS_2_/VS_2_ HS	487 K	14.7	Tunable	–	–	[51]

Theoretically predicted materials are marked by (*). The ML, BL, TL, and HS are short form of monolayer, bilayer, and heterostructure, respectively

Furthermore, a magnetoresistance (MR) effect has been detected in some graphene-based heterostructures due to the interface state in the heterostructures. Liu et al. [79] fabricated a highly stable monolayer graphene/black phosphorus (Gra/BP) heterostructure device (Fig. 7h) that exhibits a giant MR (defined as $\frac{{\left[ {R\left( B \right) - R\left( 0 \right)} \right]}}{R\left( 0 \right)} \times 100\%$) of 775% (Fig. 7i), and the nonlocal MR more than 10,000% in the Gra/BP device at room temperature due to an enhanced flavor Hall effect induced by the BP channel. Those experimental results provide valuable information for the study of magnetization dynamics in devices such as magnetoresistive random-access memories.

## Applications in Devices

### Electronic Devices

2D materials have been extensively explored as channel materials for future electronic device applications because of their atomically thin channels that offer ideal electrostatic control to enhance the immunity to the short channel effects [88] and the deprivation of band-edge sharpness resulting from chemical doping. The electronic devices based on the band engineering of 2D heterostructures have been widely reported.

**Fig. 8:**
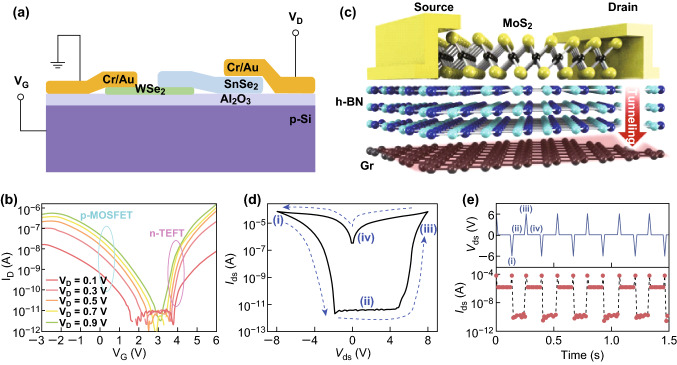
Electronic devices based on 2D heterostructure. Transistor based on the BTBT in WSe_2_/SnSe_2_ heterostructure (**a**) and its channel current (**b**) as a function of *V*_G_ at certain *V*_D_ from 0.1 to 0.9 V. Reprinted with permission from Ref. [52]. Schematic diagram of programmable floating-gate memory based on MoS_2_/hBN/graphene heterostructure (**c**), the hysteresis loop of *I*_ds_ when *V*_ds_ sweeps forward and backward (**d**) and the corresponding* I*_ds_ when periodically repeat the four states by setting *V*_ds_ to − 6, 0, and + 6 V (**e**). Reprinted with permission from Ref. [61]

Typically, the TFET usually uses an insulator thin layer of hBN as the dielectric layer, as well as TMDs with large band gap. Using 2D materials to replace traditional metallic oxides effectively avoid pinholes, oxygen doping, and interlayer defects [89]. The TFET based on the SnSe_2_/WSe_2_ vertical heterostructure (Fig. 8a) was fabricated with a subthreshold swing of 80 mV dec^−1^ and ultrahigh *I*_ON_/*I*_OFF_ ratio over 10^6^ (Fig. 8b), and such high performance can be realized simply by tuning the back-gate voltage to switch the BTBT effect [52].

Likewise, memories based on the floating-gate structure are also important applications of 2D heterostructures, where the selection of the floating-gate layer is crucial. To suppress the dark current in the device channel, gold nanoparticles (AuNPs) were selected to serve as the trapping layer [58]. Recently, researchers designed a programmable memory device based on a vertically stacked MoS_2_/hBN/graphene heterostructure with graphene as the floating-gate [61] (Fig. 8c) which has ultrahigh on/off ratio and a high stretchability (> 19%). With the dielectric layer of hBN having an appropriate thickness, the on/off ratio over 10^9^ has been obtained. Figure 8d shows the memory cycles realized by repeated voltage pulses as programming (1), reading (2), erasing (3), and reading (4) operations. Such functions can also be demonstrated in the hysteresis behavior in the *I*_ds_ − *V*_ds_ plot (Fig. 8e) and are originated from the tunneling effect through hBN and the asymmetric potential drop caused by the highly resistivity of MoS_2_. Moreover, Si et al. [90] integrated 2D ferroelectric insulator of CuInP_2_S_6_ on the top of MoS_2_ to build a nonvolatile memory device with a stable ferroelectric hysteresis loop in transfer characteristics.

### Optoelectronic Devices

**Fig. 9:**
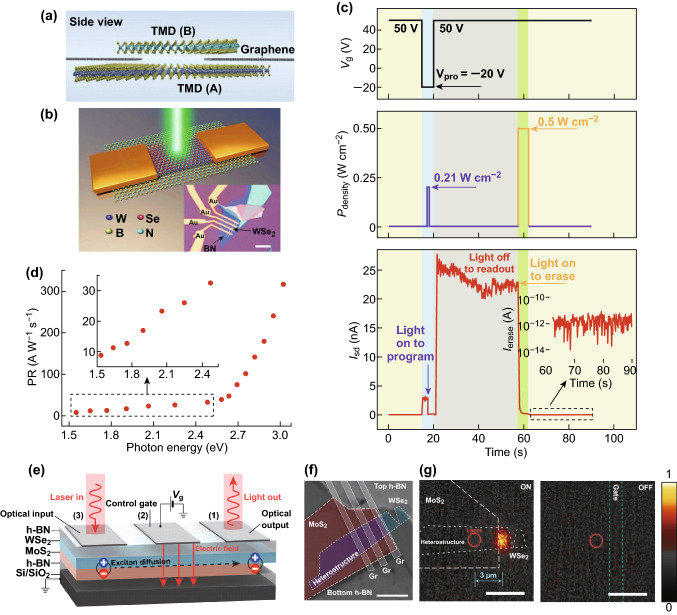
2D heterostructures in optoelectronic applications. **a** Schematic diagram of TMD/TMD heterostructure with a graphene contact. Reprinted with permission from Ref. [42]. Schematic and optical picture of optoelectronic memory based on hBN/WSe_2_ heterostructure (**b**) and the modulation of programming, reading and erasing state by *V*_g_ and incident illumination (**c**), and the wavelength-dependent PR (**d**). Reprinted with permission from Ref. [112]. Schematic and optical picture (**e**, **f**) of the multi-gate exciton device. The excitation region is marked by the red circle, and the recombination of the interlayer excitons occurs at the edge of the heterostructure. The modulation of exciton flux by *V*_g_ at room temperature is illustrated in diagram (**g**). Reprinted with permission from Ref. [113]

2D materials are outstanding candidates for optoelectronic devices due to their unique properties, including the wide response spectrum range, excellent flexibility, and strong light–matter interaction [91, 92]. Similarly, due to the generation of interlayer excitons and flexible band engineering, 2D heterostructures have been widely applied in optoelectronics including photodetectors [93, 94], photovoltaic devices, and light source devices [95–101]. Many experiments have shown that the photodetectors based on 2D heterostructures can be applied in a broad range of spectrum from ultraviolet (UV) to near-infrared (NIR), the same range as that of the photodetectors based on bulk materials, but wider than that of a single 2D heterostructure. Table 3 lists the main parameters of the latest photodetectors based on 2D heterostructures. Actually, the photodetectors based on 2D heterostructures perform better due to the generation of the strong built-in electrical field in such atomically thin structures [102]. Also, researchers are keeping exploring multi-ways to modulate devices and multi-structures to improve devices performance. It has been demonstrated that using graphene as the contact electrodes is an effective strategy to significantly increase the response speed (up to 5.5 ps Ref. [97]) of an atomically thin photodetector [95, 100, 101], because graphene, as a 2D material with ultrahigh carrier mobility, can minimize the lateral diffusion in the semiconductor [103]. Recently, Tan et al. [42] built WS_2_/MoS_2_ hetero-bilayer devices with layered graphene electrodes (Fig. 9a). By tuning the work function of graphene, the modification of the SBH at the interface of graphene and the TMD layer can be achieved. The lowering of the barrier as a result of the photogating effect would facilitate the re-injection of electrons into the TMD channel. Such devices displayed the highest photoresponsivity of up to 2340 A W^−1^ and a large internal photoconductive gain over 3.7 × 10^4^, with an estimated specific detectivity of 4 × 10^11^ Jones. Moreover, photodetectors based on MoS_2_–WS_2_ planar heterostructures [104] have also been reported to reach a detectivity of 4.36 × 10^13^ Jones. The p–n heterostructures are usually self-powered because the generated electron–hole pairs can get separated under built-in potential at zero bias. Compared with vertical heterostructures, the fabrication of in-plane junctions is much controllable and scalable due to the difficulties of controlling the stacking orientation. The extraordinary flexibility of heterostructures enabled the researchers to design a curved image sensor array based on a MoS_2_–graphene heterostructure [105] which can be applied as a human eye-inspired soft implantable optoelectronic device for detecting optical signals through programmed electrical stimulation to optic nerves.

**Table 3:** The performances of 2D-heterostructure-based photodetectors

Heterostructures	Response spectrum	Responsivity	Detectivity (Jones)	Conditions	EQE	References
MoS_2_–GaTe	–	21.83 A W^−1^	8.4 × 10^13^	*V*_g_ = 70 V	61.68%	[106]
MoS_2_/Gra/WSe_2_	400–2400 nm	10^4^ A W^−1^ (Vis)	10^11^ (NIR) 10^14^ (Vis)	*V*_ds_ = 1 V, *V*_g_ = 0 V *P*_in_ = 10^−10^ W	10^6^%	[107]
Gra/MoS_2_	–	5 × 10^8^ A W^−1^ (300 K)	–	*V*_ds_ = 0.1 V, *V*_g_ = − 50 V	–	[108]
BP/MoS_2_	–	2.17 A W^−1^	–	*V*_bias_ = 0 V	–	[109]
MoS_2_–WS_2_ planar HS	–	4.36 mA W^−1^	4.36 × 10^13^	*V*_bias_ = 0 V	1.02%	[104]
Polydiacetylene/Gra	UV to visible light	556 A W^−1^	6 × 10^11^	*V*_ds_ = 1 V, *V*_g_ = 0 V	–	[110]
Gra/WTe_2_	–	8.7 A W^−1^	–	*V*_ds_ = 0.5 V	165%	[111]
WS_2_/MoS_2_	–	1173 A W^−1^	4.1 × 10^11^	*V*_ds_ = 10 V, *V*_g_ = 0 V	–	[42]
InSe–Gra	400–1000 nm	60 A W^−1^	2.5 × 10^12^	*V*_ds_ = 10 V, *V*_g_ = 0 V	14,850%	[95]
Gra/p-GaSe/n-InSe/Gra	270–920 nm	350 A W^−1^	3.7 × 10^12^	*V*_ds_ = 2 V, *V*_g_ = 0 V	–	[100]

Moreover, the optoelectronic memories based on 2D heterostructures have been designed which can accumulate and release photo-generated carriers under an electric field and light irradiation. In addition, the introduction of 2D materials makes it possible to realize miniature, flexible, and low-energy-consumption optoelectronic storage [105]. Recently, Xiang et al. [112] successfully fabricated a multi-bit nonvolatile optoelectronic memory based on a stacked WSe_2_/hBN heterostructure as shown in Fig. 9b, which is also a filter-free color image sensor. The device can be programmed to read and erase by adjusting the gate voltage and the light pulse. The corresponding band alignment is shown in Fig. 9. The positive charges can be stored in hBN even after removing the negative gate and switching off the light (Fig. 9c). The device has performed a retention time over 4.5 × 10^4^ s and possesses over 128 (7 bit) storage states. Interestingly, the storage states at different wavelengths are highly distinct (Fig. 9d), indicating excellent wavelength distinguishing capability of the WSe_2_/hBN optoelectronic memory.

In addition, the reduced Coulomb screening in the atomically thin crystals led to a dramatic increase in the exciton binding energy and therefore stabilized these excitons at room temperature. Recently, Unuchek et al. [113] reported excitonic devices made of MoS_2_/WSe_2_ van der Waals heterostructures encapsulated in hBN (Fig. 9e) demonstrating electrically controlled multi-gate transistor actions at room temperature. The interlayer exciton was excited in the heterostructure, and the recombination was successfully observed at the edge of the heterostructure (Fig. 9g). The device was switched by adjusting the *V*_g1_, reaching an on/off ratio of 100. Furthermore, under the modulation of the bias voltage, the excitons would drift (forward bias voltage) to the low potential or be limited in the potential hydrazine (reverse bias voltage), so the diffusion distance of the exciton could be adjusted to reach 5 μm under a forward bias voltage.

### Spintronic and Valleytronic Devices

**Fig. 10:**
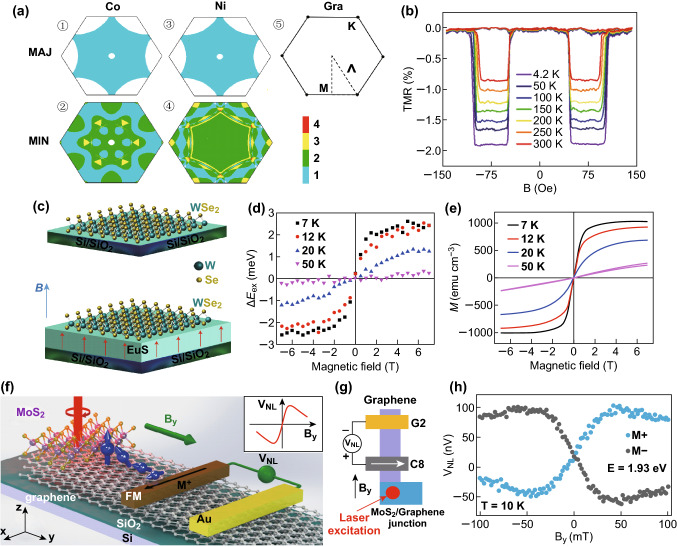
Spintronic and valleytronic applications of 2D heterostructures. **a** The calculated majority and minority spin states of Co (①–②) and Ni (③–④) and the Fermi surface of graphene (⑤), and the color bar indicates the number of Fermi surface sheets. Reprinted with permission from Ref. [118]. **b** TMR of the NiFe/Gra–hBN/Co magnetic junction at a different temperature. Reprinted with permission from Ref. [119]. WSe_2_ on the SiO_2_ substrate and the EuS substrate (**c**), and the valley splitting (**d**) and magnetization (**e**) in the WSe_2_/EuS structure under sweeping external vertical magnetic field at different temperatures. Reprinted with permission from Ref. [125]. Schematic diagram of MoS_2_/graphene heterostructure and the Hanle measurement (**f, g**) and the Hanle signal (**h**) detected after the laser excitation on the heterostructure region at a temperature of 10 K. Reprinted with permission from Ref. [126]. (Color figure online)

2D materials have excellent spin-valley properties; for example, graphene exhibits outstanding electrical, thermal, and mechanical properties. It also displays a very long spin diffusion length up to room temperature that facilitates the spin injection, manipulation, and detection in an integrated device leading to the realization of scalable and ultrafast nonvolatile logic circuits with ultralow energy dissipation. More recently, the spin valve effect in FM/2D-material/FM sandwich-like magnetic junction has been observed [114–117]. Particularly, in certain FM/G/FM junctions (FM = Ni, Co etc.), the lattice mismatch between graphene and FM is very small, and simultaneously, a spin filtering effect is theoretically permitted. As shown in Fig. 10a [118], only the spin states at Dirac point in FM can be injected into graphene and the calculation indicates that only minority spins exist at the Dirac point. The introduction of 2D heterostructures in magnetic tunneling junctions can efficiently modulate the result of the spin filter due to the different spin polarizations at different interfaces. Iqbal et al. [119] successfully observed the negative tunnel magnetoresistance (TMR) of − 0.85% in a NiFe/G–hBN/Co magnetic junction under room temperature (Fig. 10b). However, the quality of the interfaces and the lattice orientation between different layers are very crucial.

Besides, monolayer TMDs with a broken inversion symmetry own two degenerated inequivalent valleys that are related by time-reversal symmetry [120]. This property and the strong spin–orbit coupling are responsible for the unique physics in TMDs, especially the coupled spin and valley of freedom. With a direct band gap, TMDs offer the opportunity to excite carriers selectively within a particular valley with a specific valley pseudospin using circularly polarized light. Also, the valley Hall effects can be observed in a certain doped TMD sample [121, 122].

However, the external control of valleytronic devices remains a challenge because the conditions of lifting the valley degeneracy by Zeeman splitting in a single 2D material are very demanding that usually require strong magnetic field and low temperature [123], while tailoring graphene magnetic properties by structural engineering such as doping [10] and defects [124] inevitably increase the complexity in operations.

Utilizing the interfacial magnetic exchange field (MEF) from a ferromagnetic substrate that greatly enhanced valley splitting in monolayer TMDs has been found recently. Chuan Zhao et al. [125] successfully put the WSe_2_ monolayer on Si/SiO_2_ and on ferromagnetic EuS substrates. The magnetic field was perpendicular to the plane (Fig. 10c). In WSe_2_ on the SiO_2_ substrate, the Δ*E* was 1.5 meV at 7 T and 7 K. But in WSe_2_ on the EuS, the valley splitting reached 3.9 meV. Figure 10d, e shows the measurements of the systematic field and temperature dependences of the splitting. With the field increased further, the rate of Δ*E*_ex_ increase got slower and then tended to saturation at high magnetic fields. Also, with the increase in temperature, Δ*E*_ex_ decreased accordingly. Such behaviors are very similar to the field- and the temperature-dependent magnetization of EuS.

Another thought of combining is to select another material whose properties can compensate for the setbacks of the material. Typically, for the graphene and TMDs in spintronics [127], the long-distance spin transport capability of graphene has been demonstrated at room temperature [128], but the lack of the SOC made it complicated to generate pure spin current. In contrast, spin/valley polarization can be efficiently generated in monolayer TMDs such as WS_2_ and MoS_2_ via external excitation field. Recently, lateral spin or valley transport has been realized at room temperature by fabricating MoS_2_/few-layer graphene hybrid spin valves as shown in Fig. 10f. Luo et al. [126] fabricated the monolayer MoS_2_/few-layer-graphene hybrid spin valves and successfully injected the spin signal generated by circularly polarized light excitation on the TMD layer into the graphene layer. Figure 10g shows the illustration of optical spin injection, lateral spin transport, and electrical spin detection in a monolayer MoS_2_/few-layer-graphene hybrid spin valve structure. Figure 10h shows the electrical spin signal *V*_NL_ as a function of external magnetic field *B*_y_ under *T* = 10 K. The spin signal can still be easily detected at room temperature but it’s about 5 times smaller due to the increased intervalley scattering which reduces the valley polarization in monolayer MoS_2_.

## Summary and Outlook

It is clear that remarkable progress has been achieved in the study of 2D heterostructures, such as the development in realizing controllable CVD growth of planar heterostructures, the distinctive properties of magnetism and spatially separated excitons, as well as the various design and effective modulation of devices. These advances are fundamental bases for future scalable applications of 2D heterostructures. Additionally, 2D heterostructures can display unique physics phenomena due to the coupling effect and the electronic transport at the junction, offering an ideal platform for fundamental research in physics.

Nevertheless, there are still great challenges in the research of 2D heterostructures. At the first, successful synthesis of vertical heterostructures by CVD has been rare and more often the stacked heterostructures were fabricated by artificial transfer that needed a proper annealing process, a procedure that could cause damage to the samples. Thus, the priority is to develop new strategies for scalable and controllable fabrication of 2D heterostructures with high-quality interfaces and certain stack orientation, while the CVD synthesis is an ideal method its gas system needs to be optimized to for better control of the reaction conditions. As for deterministic transfer method, in-depth studies on surface physical chemistries are needed for the invention of new types of transfer carriers. Secondly, new materials, such as layered magnetic materials with high *T*_C_, need to be created, which will enable a lot of new combinations of 2D heterostructures. Thirdly, to modulate the magnetic properties, band structure, and charge transport characteristics of 2D heterostructures, conducting external electric field, optical field, magnetic field, strain treatment, together with the structural and surface engineering on the materials, will be meaningful and helpful research points. What’s more, for the fabrication and design of devices, the selection of proper materials as the electrode contact for reducing the *R*_C_ remains a tough task. The structural design of new devices to include floating-gate, multi-gate, and array structures is also very challenging.

In order to further explore the properties and applications of 2D heterostructures, several opinions could be taken into consideration for future development. Firstly, the integration of layered ferromagnetic insulator and monolayer TMD will greatly enhance the valley splitting in TMD, so the synthesis of a layered ferromagnetic insulator material whose *T*_C_ is over the room temperature could enable the development of valley-storage or valley-logical devices. Secondly, mix-dimension devices based on 0D–2D, 1D–2D, or 3D–2D heterostructures are also an effective way to solve the current problems. By combining quantum dots, nanowire, nanoribbon, or waveguide structures [129] with 2D sheets, more novel properties, such as interfacial disorders, will be realized. Finally, the fabrication strategy needs further improvement. For the CVD method, modulation of gas flow direction and temperature is a key factor for controlled reaction. For the transfer method, the development of a new transfer media layer and an automatic strategy is helpful to realize scalable fabrication and high yield. Hence, the heterostructures of 2D materials are promising for exploring new physics in 2D materials and realizing functional applications. The future of 2D heterostructures is full of massive novel possibilities.
